# Lithium batteries: Improving solid-electrolyte interphases via underpotential solvent electropolymerization

**DOI:** 10.1016/j.cplett.2016.08.045

**Published:** 2016-09-16

**Authors:** Laleh Majari Kasmaee, Asghar Aryanfar, Zarui Chikneyan, Michael R. Hoffmann, Agustín J. Colussi

**Affiliations:** Linde Center for Global Environmental Science, California Institute of Technology, Pasadena, CA 91125, USA

**Keywords:** Solid electrolyte interphase, Electropolymerization, Lithium metal batteries, Dendrite inhibition, Organic carbonates

## Abstract

Understanding the mechanism of formation of solid-electrolyte interphases (SEI) is key to the prospects of lithium metal batteries (LMB). Here, we investigate via cyclic voltammetry, impedance spectroscopy and chronoamperometry the role of kinetics in controlling the properties of the SEI generated from the reduction of propylene carbonate (PC, a typical solvent in LMB). Our observations are consistent with the operation of a radical chain PC electropolymerization into polymer units whose complexity increases at lower initiation rates. As proof-of-concept, we show that slow initiation rates via one-electron PC reduction at underpotentials consistently yields compact, electronically insulating, Li^+^-conducting, PC-impermeable SEI films.

## Highlights

• Solid-electrolyte interfaces (SEI) are polymers derived from solvent reduction.
• SEI properties depend on the degree of solvent polymerization.
• Slow reduction rates yield electronically insulating, ion-conducting, solvent-impermeable SEI films.

## Graphical Abstract

**Figure:**
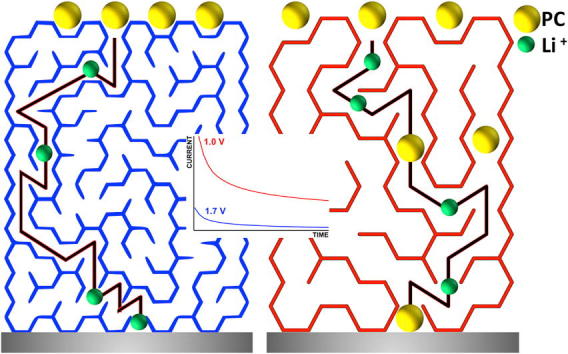

## 1 Introduction

Potentially, lithium metal batteries (LMB) are the optimal energy storage devices [1]. Li^0^, however, has an exceptional facility for growing dendrites, a feature that causes battery degradation and ultimately failure [2], [3], [4]. It has been realized that LMB prospects hinge on the inhibition of dendrite growth by the solid-electrolyte interphases (SEI) generated from the decomposition of most organic solvents at the negative potentials required to reduce Li^+^ [5].

The importance generally ascribed to SEI is most objectively attested by recent reports which emphasized that ‘…SEI formation is the most crucial and least understood phenomena impacting battery technology…’ [6], and ‘…constructing stable and efficient SEI is among the most effective strategies to inhibit the dendrite growth and achieve superior cycling performance…’ [7]. Previous attempts at improving SEI properties have variously resorted to ‘…electrolyte additives and surface modification of the cathode…(which) have been shown to improve the formation of an effective SEI layer…’ and led to the conclusion that ‘…the formation of the SEI depends largely on electrode materials, electrolyte salts, and solvents involved…’ [8]. The composition and structure of SEI have also been intensively investigated by diverse techniques, such as XPS, solid state NMR [9], ellipsometry [10], sum-frequency generation spectroscopy [11], electron microscopies [12], neutron scattering [13], AFM [14], electron paramagnetic spectroscopy and matrix assisted laser desorption ionization (MALDI) time of flight mass spectrometry [15].

Recent reviews, however, have acknowledged that ‘…many strategies have been proposed to modify SEI structure. However, the modifying process is still out of control in a bulk cell because the thickness, density and ion conductivity cannot yet be rationally designed’ [7]. One interpretation of this impasse is that SEI properties depend not only on initial conditions, such as electrode materials, electrolyte salts, solvents and additives, but on the procedure by which SEI are generated. Thus, if the mechanisms of generation that would allow us to rationally design SEI are still elusive it is simply because mechanisms cannot be deduced from information on initial and final states alone. Here, we address this issue in an experimental study of the kinetics of electropolymerization of propylene carbonate (PC) into SEI on metal cathodes [16], [17], in conjunction with a fundamental analysis of the results obtained. Our goal was to gain insight into the mechanism of SEI generation.

## 2 Results and discussion

### 2.1 Cyclic voltammetry

**Fig. 1:**
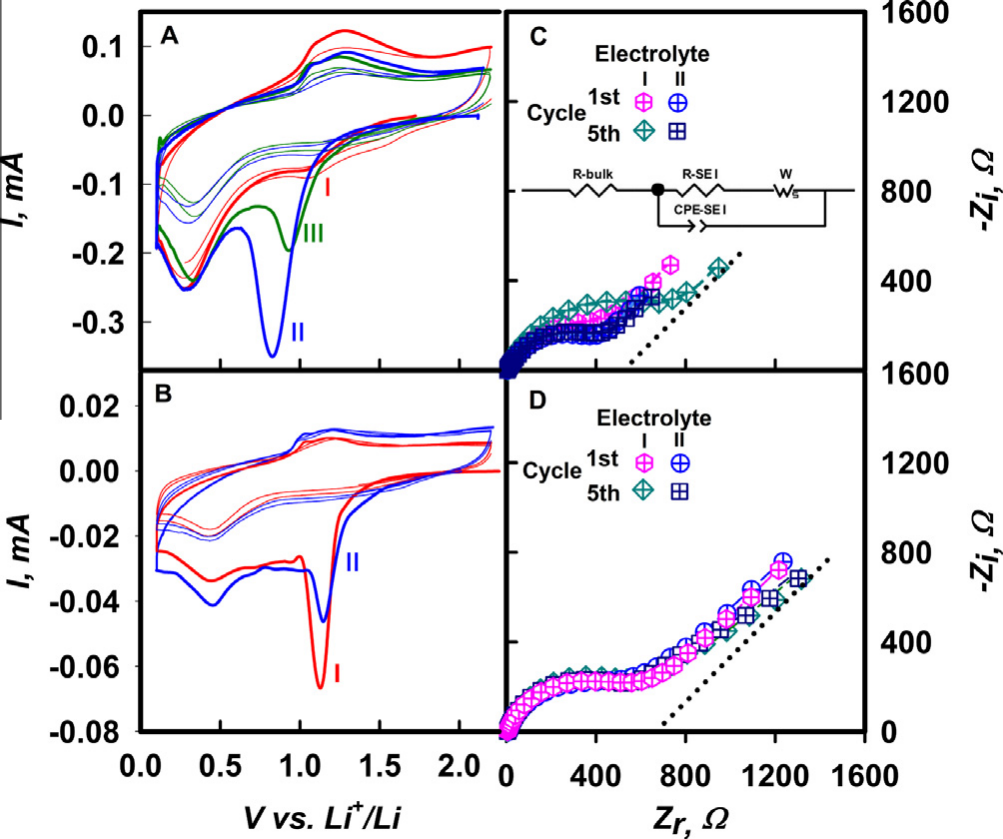
Cyclic voltammograms in Cu|electrolyte|Li cells filled with electrolytes **I** (1 M LiClO_4_), **II** (0.1 M LiF + 0.9 M LiClO_4_) and **III** (0.01 M LiF + 0.99 M LiClO_4_), as indicated. **A**: scanned at *v* = 5 mV s^−1^. **B**: at *v* = 0.5 mV s^−1^; Open circuit voltage (OCV) Nyquist diagrams of cells after undergoing CV scans between OCV and 0.1 V (vs 1 M Li^+^/Li^0^ in PC) **C**: at *v* = $5\;\text{mV}\;{\text{s}}^{-1}$; Inset: equivalent circuit. **D:** at *v* = 0.5 mV s^−1^. Fitting parameters in Table 2A, Table 2B. Dotted lines: Warburg’s *n*′ = 0.5 slopes as a reference, see text.

We began probing the electrochemical characteristics of our coin LMB (See Experimental Methods) by running single *v* = 5 mV s^−^^1^ cathodic scans in 1 M LiClO_4_ (**I**) and [0.1 M LiF + 0.9 M LiClO_4_] (**II**) electrolytes (Table 1) from open circuit voltage (OCV) down to 0.1 V (hereafter, all voltages relative to 1 M Li^+^/Li^0^ in PC). We chose ClO_4_^−^ because it is a stable, weakly coordinating anion [18], and F^−^ as additive because it is known to improve LMB performance [2], [19]. PC is widely used as the base solvent in LMB, to which other organic carbonates are usually added to improve its performance [20], [21]. In Fig. 1A and B, peaks between 0.8 and 1.0 V correspond to PC reduction (PCR hereafter) [22]. Peaks at 0.3 V are assigned to the underpotential deposition (UPD) of Li^0^ on the basis of similar peaks within 0.4–0.6 V reported in the literature [23], and the fact that the peak at 1.3 V associated with the anodic stripping of UPD Li^0^ deposits does not appear following cathodic scans that were reversed at 0.9 V to avoid Li^0^ deposition.

The areas under PCR peaks from electrolytes **I**, **II** and **III** (uncorrected for small capacitive currents) correspond to the circulation of Q_I_ ∼ 3, Q_II_ = 15 and Q_III_ = 6 mC cm^−^^2^ respectively. From Fig. 1A we learn that: (1) PCR is irreversible in all electrolytes, i.e., leads to products that are not oxidized below 2 V, (2) the presence of F^−^ significantly increases PCR peak intensities, (3) PCR peaks produced in the first scan from electrolytes **II** and **III** do not reappear in successive scans, at variance with the weaker PCR peaks from electrolyte **I**. To interpret these findings, it is important to realize that although PCR (reaction R1 below) formally takes place in the presence of excess PC in all cases, in fact strongly dipolar PC (dipole moment = 4.9 Debye) is not freely available as such because it is strongly bound to Li^+^ as Li(PC)_n_^+^ (See Li(PC)_n_^+^ desolvation notes, SI) [24], [25]. Therefore, the dissimilar faradaic charges Q associated with PCR in the absence vs the presence of F^−^ suggest that, (1) PCR is kinetically limited by the availability of free PC in electrolyte **I** at *v* = 5 mV s^−^^1^, i.e., the desolvation process that releases PC from Li(PC)_n_^+^ is rate controlling under such conditions, (2) Li(PC)_n_^+^ desolvation is catalyzed by F^−^.

The role and effectivity of F^−^ in catalyzing the release of PC are evidenced by the significant shift of peak potentials in **II** and **III** to less positive values than in **I**, i.e., to potentials at which PCR can compete with the F^−^-catalyzed Li(PC)_n_^+^ desolvation [26]. Together, these observations imply that PCR at 5 mV s^−^^1^ is preceded and followed by slow chemical reactions, i.e.: that under such conditions SEI generation takes place by a CEC mechanism [27]. If the above analysis were true, Li(PC)_n_^+^ desolvation should cease to be rate controlling at sufficiently slow charging conditions, as shown below.

The role of homogeneous kinetics on the formation and properties of SEI layers was fully confirmed by voltammograms carried at tenfold slower *v* = 0.5 mV s^−^^1^ scan rates (Fig. 1B). In this case, first-scan PCR peaks occur at similar potentials and have similar intensities from 1 M LiClO_4_ (**I**) and [0.1 M LiF + 0.9 M LiClO_4_] (**II**) electrolytes. Moreover, none reappear in successive scans. Noteworthy, and mechanistically meaningful (see below), is that PCR peaks are narrower and shift to more positive potentials than those observed at *v* = 5 mV s^−^^1^, thereby implying that PCR is now controlled by a different process. These experiments clearly show that F^−^ is not essential because compact layers can also be generated in the absence of F by scanning at slower rates. The nearly identical Faradaic charges that circulate during PCR from **I** and **II** at 0.5 mV s^−^^1^: Q_I_ ∼ 13 mC, Q_II_ ∼ 12 mC cm^−^^2^ (from Fig. 1B), vs. Q_I_ ∼ 3, Q_II_ ∼ 15 mC cm^−^^2^ at 5 mV s^−^^1^ (from Fig. 1A) confirm that PCR at 5 mV s^−^^1^ scan rates in the absence of F^−^ is kinetically limited by the release of PC from Li(PC)_4_^+^.

### 2.2 Electrochemical impedance spectroscopy

The electrical characteristics of the SEI produced in the experiments of Fig. 1A and B were analyzed by electrochemical impedance spectroscopy (EIS). Note that our EIS experiments were performed at open circuit voltage (OCV), i.e., they only provide information about the static electrical properties of preformed SEI. Fig. 1C shows Nyquist diagrams of the SEI produced after *v* = 5 mV s^−^^1^ cathodic scans from **I** and **II**. All diagrams consist of a single depressed semicircle at medium and high frequencies, which merges at low frequencies into a straight line associated with Li^+^ diffusion through SEI layers. The presence of a single semicircle excludes significant contributions from multiple SEI layers, i.e., SEI properties can be accounted by a single layer despite their complex, heterogeneous morphology and chemical composition [15]. We analyzed these spectra in terms of the equivalent electrical circuit shown in the inset of Fig. 1C. *R-bulk* is the sum of ohmic drops across the electrolyte and other cell components, *R-SEI* and *CPE-SEI* are the resistance and capacitance of preformed SEI layers, and *W* is the impedance arising from Li^+^ diffusion through SEI layers (Table 2A) [26].

The relevant features of Fig. 1C are: (1) the Nyquist diagram for the SEI produced from **II** after the 5th cycle overlaps that registered after the 1st cycle, in contrast with those for the SEI produced from **I** (in the absence of F^−^), and (2) SEI layers produced from **I** keep increasing through the 5th cycle, i.e., they are permeable to PC. Key entries in Table 2A are the resistances R-SEI of SEI layers grown in first cycles from **I** and **II**. They are informative because R-SEI have comparable values despite the fact that the amount of PC reduced from electrolyte **I** is only 1/5th of that reduced from electrolyte **II:** Q_I_ ∼ 3 vs Q_II_ ∼ 15 mC cm^−^^2^. The implication is that SEI production may be initiated by PCR, but is surely followed by homogeneous chemical reactions that incorporate substantial amounts of additional PC into the layers.

Constant phase element (CPE) Z_r_ vs ω plots, Eq. (E1)

(E1) $${\text{Z}}_{\text{r}}={\text{Z}}_{0}+\sigma \;\omega^{-n^{\prime }}$$

where *Z_r_* is the real impedance, *Z*_0_ is a constant, ω is frequency, and σ and *n*′ are adjustable parameters, led to *n*′ ∼ 0.6 values that are significantly larger that the *n*′ = 0.5 value expected for Warburg’s impedance associated with Li^+^ diffusion in semi-infinite, homogeneous SEI layers. A detailed analysis of the physical underpinnings of *n*′ > 0.5 values is beyond the scope of this paper [28], but it is noteworthy that Warburg’s *n*′ = 0.5 value does occur in SEI layers produced at lower scan rates (see below and Table 2B). This finding represents evidence that the morphology of SEI layers is sensitive to scan rates and/or PCR rates, i.e., to the kinetics of SEI formation. Li^+^ diffusion coefficient, *D*_Li_^+^, in the SEI formed was obtained from Eq. (E2) [26].

(E2) $$D_{\mathrm{Li}}^{+}=\frac{R^{2}T^{2}}{2\theta^{2}n^{4}F^{4}{\left[{\mathrm{Li}}^{+}\right]}^{2}\sigma^{2}}$$

where *R* is the gas constant, *T* is absolute temperature (298 K), *θ* is electrode area, *n* = 1 is the ion charge, *F* is Faraday’s constant, and σ is the slope of Z_r_ vs ω^−^^n′^ plots. The *D*_Li_^+^ ∼ 10^−^^13^ cm^2^ s^−^^1^ values we derive correspond to Li^+^ transport through SEI layers, which are compatible with the *D*_Li_^+^ ∼ 10^−^^12^ cm^2^ s^−^^1^ values reported for porous SEI (vs. *D*_Li_^+^ ∼ 10^−^^16^ cm^2^ s^−^^1^ in compact SEI) [29]. They should not be mistaken with the larger *D*_Li_^+^ ∼ 10^−^^10^ cm^2^ s^−^^1^ diffusivities measured in porous graphite electrodes [30]. The resistance of SEI layers is directly proportional to thickness *l*, and electrical resistivity, *ρ*, Eq. (E3):

(E3) $$R\text{-}\mathrm{SEI}=\frac{\rho l}{\theta }$$

In marked contrast with the above results, the Nyquist diagrams of SEI grown at *v* = 0.5 mV s^−^^1^ (Fig. 1D) are qualitatively and quantitatively different from those in Fig. 1C. In this case, the SEI produced from electrolytes **I** and **II** after the 1st and 5th scans have essentially identical parameters (Table 2B), which are consistent with electronically insulating and PC-impermeable SEI layers.

**Fig. 2:**
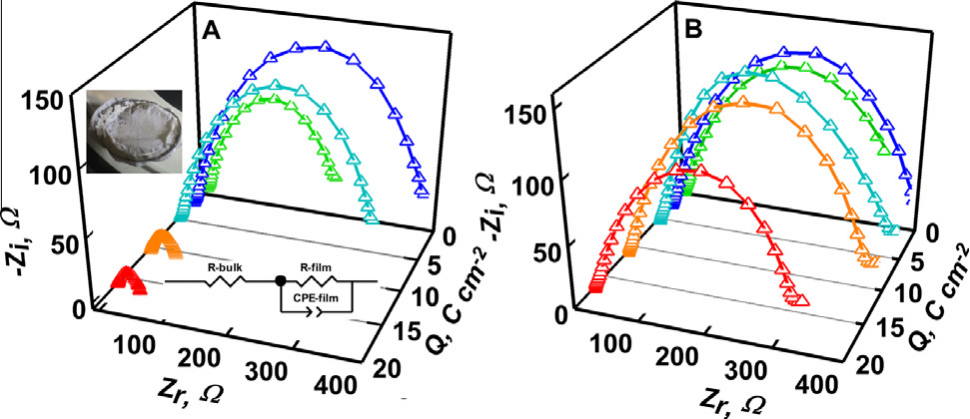
Nyquist diagrams of Li|electrolyte|Li symmetrical cells at open circuit voltage after being charged galvanostatically at 0.05 mA cm^−2^ for variable periods. A: cells filled with electrolyte **I**. B: cell filled with electrolyte **II**. A insets: (1) the assumed equivalent circuit, (2) a picture of lithium dendrites that short-circuited the cell.

The presence of fluoride has a significant effect on the long-term stability of batteries upon galvanostatic charging at 0.05 mA cm^−^^2^. Note that PC and Li^+^ are simultaneously reduced during galvanostatic charging. Fig. 2A and B show the evolution of Nyquist diagrams as functions of circulated charge. Noteworthy is the fact that the resistance of cells filled with **II** (containing F^−^) decreases by only ∼25% after the circulation of Q > 17 C cm^−^^2^, whereas the resistance of cells filled with **I** (without F^−^) already drops eightfold at Q > 5 C cm^−^^2^, as an indication that Li^0^ dendrites had pierced SEI layers, reached the cathode and short-circuited the battery. Fluoride additions also enhance the persistence of SEI layers (See Fig. S1, SI).

### 2.3 Chronoamperometry

**Fig. 3:**
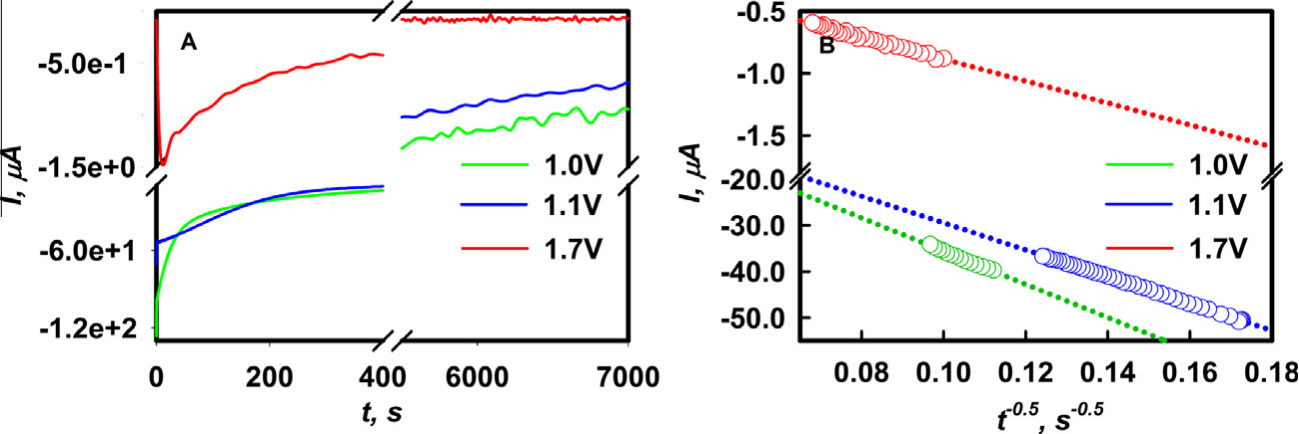
**A:** Chronoamperograms in Cu|electrolyte|Li cells filled with electrolyte **I** under 1.0 V, 1.1 V and 1.7 V applied voltages (vs 1 M Li^+^/Li^0^ in PC). **B:** Cottrell current*I*vs. (time)^−½^ plots (Eq. (E4)).

The finding that PCR at slow scan rates generates PC-impermeable SEI layers (Fig. 1C and D) led us to test the dependence of PCR rates on applied potential by growing SEI under potentiostatic conditions in chronoamperometric (CA) experiments. CA experiments at 1.0, 1.1 and 1.7 V (vs Li^+^/Li^0^) applied potentials in cells filled with electrolyte **I** are shown in Fig. 3A. Faradaic currents associated with PCR (i.e., those circulating after the decay of initial capacitive currents) markedly increase at more negative overpotentials: η = V − E_p_ (PCR rates peak at E_p_ ∼ 1.3 V, Fig. 3B), as expected. Confirming our expectation that slow PCR rates would lead to self-healing SEI layers, currents circulating in the CA at a η ∼ 1.7–1.3 V = 0.4 V underpotential vanish after ∼5000 s, in contrast with experiments carried at 1.0 V and 1.1 V. Past the initial stages where currents are partially due to the capacitive charging of double layers (and also at *i* *>* 50 μA, partially controlled by PC desolvation, cf. Fig. 1A and B), the slopes of faradaic currents vs (time)^−½^ (Fig. 3B), Eq. (E4):

(E4) $$i=\frac{\mathrm{nF}\theta [\mathrm{PC}]\sqrt{D_{\mathrm{PC}}}}{\sqrt{\pi t}}$$

lead to vastly different PC diffusion coefficients in SEI layers grown at 1.0 V: *D*_PC_(1.0 V) = 8.3 × 10^−^^14^ cm^2^ s^−^^1^ vs. those grown at 1.7 V: *D*_PC_(1.7 V) = 7.7 × 10^−^^17^ cm^2^ s^−1^, which are compatible with the *D*_PC_ ∼ 10^−12^ to 10^−16^ cm^2^ s^−1^ values reported in porous and compact SEI layers, respectively [29]. Note that Eq. (E4) for PC diffusion through a growing solid SEI layer is the analogue of Cottrell’s equation for ion diffusion through a widening, solvent-filled double layer. In both cases layer thicknesses increase with t^½^, and the corresponding current densities decrease with t^−½^ [26]. Most remarkably, *D*_PC_(1.7 V) is ∼1100 times smaller than *D*_PC_(1.0 V) through SEI layers that were seeded by a small fraction of the charge: Q_1.7V_/Q_1.0V_ = 0.65 mC cm^−2^/17.5 mC cm^−2^ = 0.04 (Q′s are the areas under *i* vs t plots), underscoring that dramatic impact of slow PCR rates on SEI compactness.

**Fig. 4:**
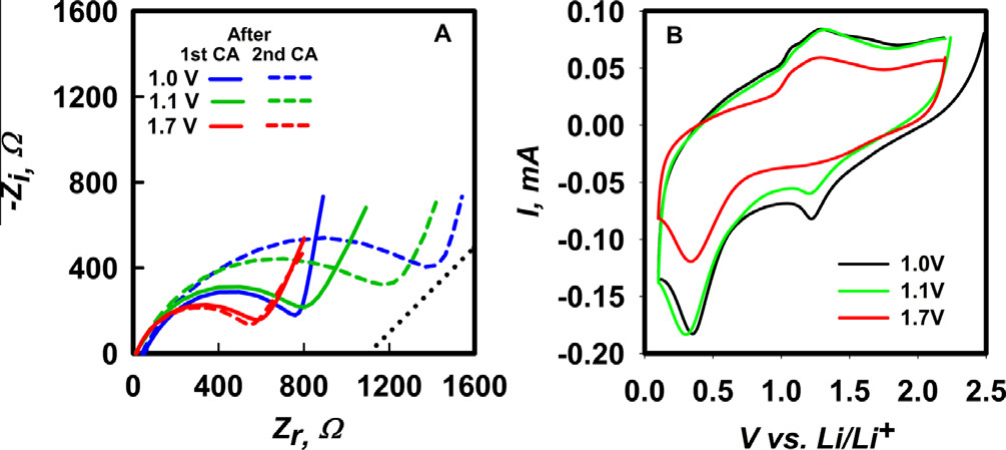
**A:** Nyquist diagrams at open circuit voltage of Cu|electrolyte|Li cells filled with electrolyte **I** after the first and second chronoamperometries at 1.0 V, 1.1 V and 1.7 V applied voltages vs. 1 M Li^+^/Li^0^ in PC. Dotted line: Warburg’s *n*′ = 0.5 slope as a reference. **B**: Cyclic voltammograms at *v* = 5 mV s^−1^ in cells filled with electrolyte **I** after being charged potentiostatically at 1.0 V, 1.1 V and 1.7 V for 2 h.

The relevant electrochemical characteristics of the SEI layers grown potentiostatically were probed by EIS and CV experiments. Nyquist diagrams of the SEI layers produced in successive chronoamperometry experiments under 1.0, 1.1 and 1.7 applied potentials are shown in Fig. 4A. The parameters derived from their analysis are compiled in Table 3. It is apparent that the SEI produced in the first 1.7 V potentiostatic experiment does not grow upon further charging, in contrast with those produced at 1.0 and 1.1 V. This conclusion is corroborated by CV scans (Fig. 4B).

Inspection of Table 3 reveals that (1) R-SEI remains nearly constant in 1.7 V experiments but increases by a factor of 2 in the second CA at 1.0 V, (2) C-SEI of the SEI layer produced at 1.7 V is about 2 times larger than those at 1.0 and 1.1 V suggesting (since C ∝ 1/thickness) that they are about half as thick, and (3) Li^+^ diffusion, with *n*′ > 0.7 > 0.5, is anomalous in all cases. Noteworthy is that *D*_Li_^+^ for SEI layers produced at 1.7 V is comparable to the *D*_Li_^+^ values in the SEI obtained in potentiodynamic CV experiments (see Table 2A, Table 2B), but much smaller than *D*_Li_^+^ in SEI layers produced at 1.0 and 1.1 V, as evidence that SEI morphology is a sensitive function of applied potentials. Summing up, the above findings are consistent with (1) SEI layers that incorporate PC molecules in larger numbers than those undergoing reduction at the electrode surface, i.e., SEI are essentially polymer materials [31], and (2) SEI properties strongly depend on the kinetics of the generation process [32].

Since SEI behave as polymeric materials, our findings suggest that the potential impact of experimental conditions on their properties should be evaluated on the basis of polymer science concepts [33], [34]. What to expect for SEI generated in a polymerization process initiated by PC reduction, reaction R1? [35], [36]:

(R1) $$\text{PC}+{\text{e}}^{-}\to {\text{PC}}^{-}$$

Following previous reports [31], [37], [38], PC^•−^ is deemed to open its ring into an alkoxycarbonyl radical followed by decomposition into CO or CO_2_, plus simpler radical anions, X^•−^. X^•−^ initiate radical (or anionic; see Polymerization notes in SI) chain-growth polymerizations propagated by reactions R2:

(R2) $${\text{X}}^{-}+\text{PC}\to \text{X-}{\left(\text{PC}\right)}^{-}\to \to \text{X-}{\left(\text{PC}\right)}_{\text{n}}^{-}$$

and terminated via bimolecular radical recombination, reaction R3 [6],

(R3) $$\text{X-}{\left(\text{PC}\right)}_{\text{n}}^{-}+\text{X-}{\left(\text{PC}\right)}_{\text{m}}^{-}\to {\text{X}}_{2}{\left(\text{PC}\right)}_{\text{n}+\text{m}}^{2-}$$

SEI permeability, ionic and electronic conductivity, solubility and mechanical properties are essentially determined by the degree of solvent polymerization λ, i.e., by the number of monomers incorporated into polymer units [37], [38]. λ is controlled by the competition between radical propagation (R2) vs. radical termination (R3), Eq. (E5):

(E5) $$\lambda =\frac{k_{2}[{\mathrm{PC}}^{-}][\mathrm{PC}]}{2k_{3}{\left[{\mathrm{PC}}^{-}\right]}^{2}}=\frac{k_{2}[\mathrm{PC}]}{2k_{3}[{\mathrm{PC}}^{-}]}$$

where *k*′s are bimolecular reaction rate constants. Because initiation rates *r_i_* and termination rates balance at steady state, Eq. (E6):

(E6) $$r_{i}=2\;k_{3}{\left[{\mathrm{PC}}^{-}\right]}^{2}\;i.e.:\;[{\mathrm{PC}}^{-}]={\left(\frac{r_{i}}{2k_{3}}\right)}^{0.5}$$

We arrive at Eq. (E7):

(E7) $$\lambda =\frac{k_{2}[\mathrm{PC}]}{\sqrt{2k_{3}r_{i}}}$$

which predicts that the degree of polymerization should be directly proportional to PC concentration at the front of advancing radical chains, and inversely proportional to (initiation rates: *r_i_* = *r*_1_)^½^. The fundamental λ ∝ *r*^−½^ relationship for a radical chain polymerization should apply whether Li^+^ are present in the SEI, as in the present case, or not.

Thus, our CA experiments at 1.7 V are deemed to produce functional SEI layers because the low current densities exclusively associated with PC reduction provide the slow initiation rates required to generate long polymerization chains. Furthermore, as a result, the overall slow polymerization process they bring about may not be limited by the availability of the free PC monomers released from the slow desolvation of Li(PC)_n_^+^. The very low value of the PC diffusion coefficient *D*_PC_(1.7 V) = 7.7×10^−17^ cm^2^ s^−1^ determined in the SEI generated at underpotential is clearly consistent with transport through a compact material comprising few, long and possibly linked or intertwined polymer chains [29]. From this perspective, the PC reduction rates at the ∼1 V overpotentials prevailing under conventional LMB charging conditions, where the full voltage required to plate the anode is applied from the onset, may not be ideal because they are likely to generate short, disjoint polymer domains rather than compact, interconnected polymer films extending over the electrode surface. We believe that our results and analysis provide new insights into the outstanding questions formulated in a recent review on the subject: ‘how does SEI form?’ and ‘what parameters control SEI properties?’ [39].

## 3 Experimental methods

Experiments were performed in two types of electrochemical cells filled with electrolyte solutions **I**, **II** and **III** of three different compositions (Table 1). Studies on SEI layers were carried out in Cu|electrolyte|Li coin cells whereas the deposition of Li^0^ films was investigated in Li|electrolyte|Li coin cells. Round disk electrodes (area = θ = 1.6 cm^2^) were punched from Li^0^ foil (Aldrich, 99.9%, 0.38 mm thick) that had been polished by scraping with a blade and rinsed with dimethyl carbonate. Electrodes were mounted on a transparent poly-methyl methacrylate separator that kept them *L* = 3.175 mm apart. All operations were carried out in a glove box sparged with argon. Chronoamperometry (CA), electrochemical impedance spectroscopy (EIS) and cyclic voltammetry (CV) measurements were made with a Bio-Logic VSP potentiostat. Galvanostatic experiments were performed with an ARBIN BT2000 battery tester. EIS experiments (5 mV modulation signal amplitude) covered the 100 mHz to 1 MHz frequency range. Impedance data were analyzed using Zview software. All reported potentials are relative to Li^+^/Li^0^ under working conditions. Li^0^ and Cu^0^ foils (Sigma-Aldrich) were used as-received. Lithium perchlorate (LiClO_4_, Aldrich, battery grade, 99.99%) and lithium fluoride (LiF, Aldrich, 99.99% trace metal basis) were dried at 90 °C under vacuum for 24 h and dissolved in propylene carbonate (PC) (Aldrich, 99.7% anhydrous). Further details can be found in previous publications from our laboratory [2], [3], [40], [41].

## Conflict of interest

The authors declare no competing financial interests.

## Figures and Tables

**Table 1:** Electrolyte compositions.

**I:**	1.00 M LiClO_4_
**II:**	0.10 M LiF + 0.90 M LiClO_4_
**III:**	0.01 M LiF + 0.99 M LiClO_4_

**Table 2A:** Equivalent circuit parameters from the impedance spectra of Fig. 1C.

CV cycle	Electrolyte	*R-Bulk* (Ω)	*R-SEI* (Ω)	*C-SEI* (10^5^ Ω s^n^)	*n*	*σ* (10^4^ Ω^−1^ s^n′^)	*n*′	$D_{{\mathrm{Li}}^{+}}$ (10^13^ cm^2^ s^−1^)
1st	**I**	4	543	1.3	0.74	2.1	0.62	3.0
	**II**	3.1	459	1.5	0.73	3.6	0.60	1.1

5th	**I**	4.7	846	1	0.76	2.1	0.67	2.9
	**II**	3	489	1.6	0.72	3.9	0.62	0.9

**Table 2B:** Equivalent circuit parameters from the impedance spectra of Fig. 1D.

CV cycle	Electrolyte	*R-Bulk* (Ω)	*R-SEI* (Ω)	*C-SEI* (10^5^ Ω s^n^)	*n*	*σ* (10^4^ Ω^−1^ s^n′^)	*n*′	$D_{{\mathrm{Li}}^{+}}$ (10^13^ cm^2^ s^−1^)
1st	**I**	5.3	549	2.7	0.79	3.2	0.50	1.3
	**II**	4.3	503	2.9	0.79	3.3	0.49	1.2

5th	**I**	4.7	543	3.1	0.82	3.0	0.46	1.5
	**II**	5.6	534	2.8	0.82	3.2	0.45	1.3

**Table 3:** Equivalent circuit parameters for the impedance spectra of Fig. 4A.

Applied voltage V vs Li^+^/Li^0^	CA	*R-Bulk* (Ω)	*R-SEI* (Ω)	*C-SEI* (10^5^ Ω s^n^)	*n*	*σ* (10^4^ Ω^−1^s^n′^)	*n*′	$D_{{\mathrm{Li}}^{+}}$ (10^13^ cm^2^s^−1^)
1.0	1st	30	800	0.89	0.77	0.35	0.85	110
	2nd	35	1641	0.77	0.72	0.34	0.90	120

1.1	1st	14	920	0.95	0.73	0.79	0.78	21
	2nd	15	1336	0.72	0.73	0.71	0.77	26

1.7	1st	16	616	1.8	0.79	1.4	0.73	7.3
	2nd	16	565	1.6	0.80	2.0	0.71	3.4

## References

[1] Palacin M.R., de Guibert A. (2016). Science.

[2] Aryanfar A., Brooks D., Merinov B.V., Goddard W.A., Colussi A.J., Hoffmann M.R. (2014). J. Phys. Chem. Lett..

[3] Aryanfar A., Cheng T., Colussi A.J., Merinov B.V., Goddard W.A., Hoffmann M.R. (2015). J. Chem. Phys..

[4] Chen Q., Geng K., Sieradzki K. (2015). J. Electrochem. Soc..

[5] Zhang Y.H., Qian J.F., Xu W., Russell S.M., Chen X.L., Nasybulin E., Bhattacharya P., Engelhard M.H., Mei D.H., Cao R.G., Ding F., Cresce A.V., Xu K., Zhang J.G. (2014). Nano Lett..

[6] Soto F.A., Ma Y.G., de la Hoz J.M.M., Seminario J.M., Balbuena P.B. (2015). Chem. Mater..

[7] Cheng X.-B., Zhang R., Zhao C.-Z., Wei F., Zhang J.-G., Zhang Q. (2015). Adv. Sci..

[8] Agubra V.A., Fergus J.W. (2014). J. Power Sources.

[9] Blanc F., Leskes M., Grey C.P. (2013). Acc. Chem. Res..

[10] Lux S.F., Lucas I.T., Chevalier J.S., Richardson T.J., Kostecki R., Kostecki R., Abe T., Liaw B.Y. (2012). Interfaces and Interphases in Battery Systems.

[11] Yu L., Liu H.J., Wang Y., Kuwata N., Osawa M., Kawamura J., Ye S. (2013). Angewandte Chemie-Int. Ed..

[12] Lee S.H., You H.G., Han K.S., Kim J., Jung I.H., Song J.H. (2014). J. Power Sources.

[13] Bridges C.A., Sun X.G., Zhao J.K., Paranthaman M.P., Dai S. (2012). J. Phys. Chem. C.

[14] Lu Y.Y., Tu Z.Y., Archer L.A. (2014). Nat. Mater..

[15] Xu K. (2014). Chem. Rev..

[16] Osaka T., Naoi K., Ogano S., Nakamura S. (1987). J. Electrochem. Soc..

[17] An S.J., Li J.L., Daniel C., Mohanty D., Nagpure S., Wood D.L. (2016). Carbon.

[18] Mauro V., D’Aprano A., Croce F., Salomon M. (2005). J. Power Sources.

[19] Choudhury S., Archer L.A. (2016). Adv. Elect. Mater..

[20] Nie M.Y., Demeaux J., Young B.T., Heskett D.R., Chen Y.J., Bose A., Woicik J.C., Lucht B.L. (2015). J. Electrochem. Soc..

[21] Profatilova I.A., Stock C., Schmitz A., Passerini S., Winter M. (2013). J. Power Sources.

[22] Schäffner B., Schäffner F., Verevkin S.P., Börner A. (2010). Chem. Rev..

[23] Saito T., Uosaki K. (2003). J. Electrochem. Soc..

[24] Ohtani H., Hirao Y., Ito A., Tanaka K., Hatozaki O. (2010). J. Therm. Anal. Calorim..

[25] Xu K. (2004). Chem. Rev.-Columb..

[26] Bard A.J., Faulkner L.R. (2000). Electrochemical Methods: Fundamentals and Applications.

[27] Bureau C. (1999). J. Electroanal. Chem..

[28] Bisquert J., Compte A. (2001). J. Electroanal. Chem..

[29] Guan P., Liu L., Lin X. (2015). J. Electrochem. Soc..

[30] Park M., Zhang X., Chung M., Less G.B., Sastry A.M. (2010). J. Power Sources.

[31] Tavassol H., Buthker J.W., Ferguson G.A., Curtiss L.A., Gewirth A.A. (2012). J. Electrochem. Soc..

[32] Koh J.Y., Jung Y. (2013). Int. J. Electrochem. Sci..

[33] Flory P.J. (1953). Principles of Polymer Chemistry.

[34] Karyakin A., Cosnier S. (2010). Electropolymerization: Concepts, Materials and Applications.

[35] Seo D.M., Chalasani D., Parimalam B.S., Kadam R., Nie M.Y., Lucht B.L. (2014). ECS Electrochem. Lett..

[36] Gachot G., Grugeon S., Armand M., Pilard S., Guenot P., Tarascon J.M., Laruelle S. (2008). J. Power Sources.

[37] Shkrob I.A., Zhu Y., Marin T.W., Abraham D. (2013). J. Phys. Chem. C.

[38] Shkrob I.A., Zhu Y., Marin T.W., Abraham D. (2013). J. Phys. Chem. C.

[39] Gauthier M., Carney T.J., Grimaud A., Giordano L., Pour N., Chang H.H., Fenning D.P., Lux S.F., Paschos O., Bauer C., Magia F., Lupart S., Lamp P., Shao-Horn Y. (2015). J. Phys. Chem. Lett..

[40] Aryanfar A., Brooks D.J., Colussi A.J., Hoffmann M.R. (2014). Phys. Chem. Chem. Phys..

[41] Aryanfar A., Brooks D.J., Colussi A.J., Merinov B.V., Goddard W.A., Hoffmann M.R. (2015). Phys. Chem. Chem. Phys..

