# Supplementary material for: Lithium batteries: Improving solid-electrolyte interphases via underpotential solvent electropolymerization
Source: Chem Phys Lett. 2016 Sep 16;661:65–9. doi: 10.1016/j.cplett.2016.08.045 (PMC5063536; doi:10.1016/j.cplett.2016.08.045)
Supplement: Supplementary data 1 [file mmc1.pdf]

Supporting Information

Lithium batteries: Improving solid-electrolyte interphases via underpotential solvent electropolymerization

*Laleh Majari Kasmaee, Asghar Aryanfar, Zarui Chikneyan, Michael R. Hoffmann and Agustín J. Colussi**

Linde Center for Global Environmental Science, California Institute of Technology, Pasadena, CA, 91125

The Nyquist diagrams of Fig. S1, which show the impedance of fresh cells filled with **I** and **II** before and immediately after being charged, and after a one-day aging period. The SEI produced in the absence of F^- nearly disappeared after 1 day, in contrast with the limited dissolution of the SEI produced in its presence. These findings are in line with previous reports on fluoride effects.[1,2]

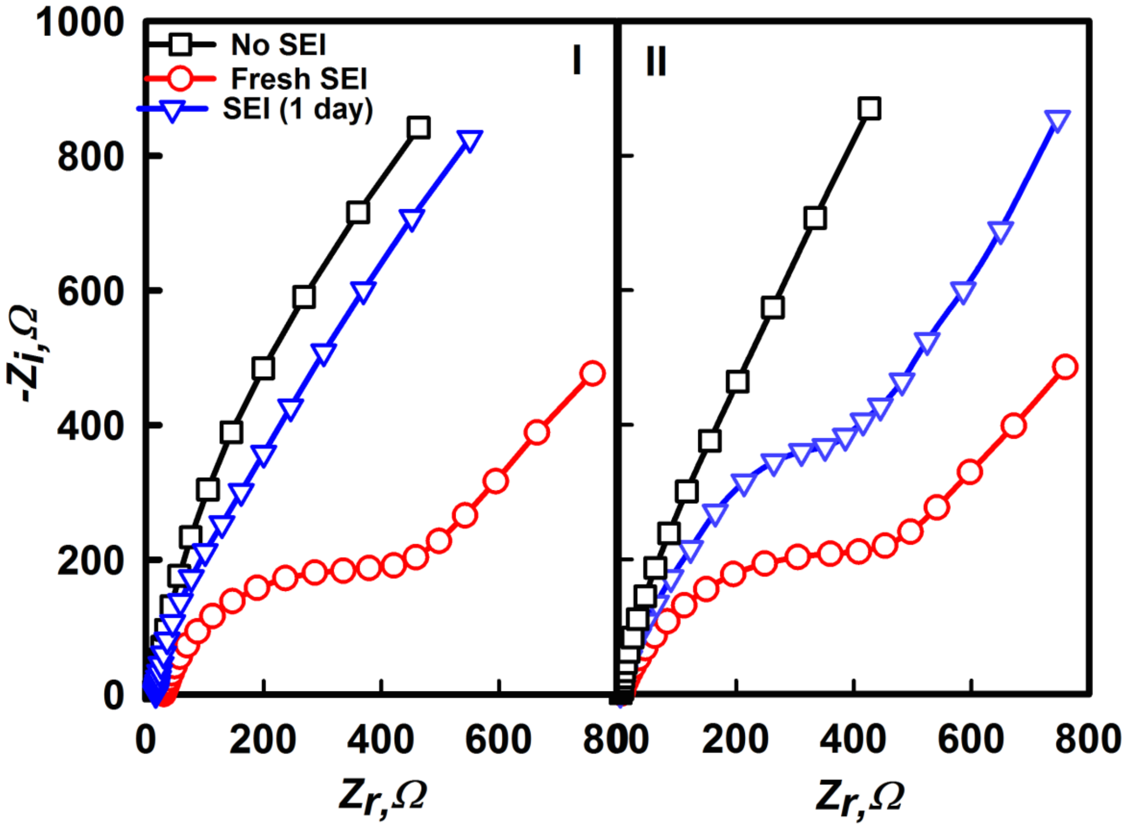

Figure S1. Nyquist diagrams at open circuit voltage of Cu|electrolyte|Li cells filled with electrolytes **I** and **II** recorded in pristine cells (no SEI), immediately after being charged (fresh SEI), and after a one-day aging period (SEI 1 day), as indicated in the figure.

$Li(PC)_n^+$ desolvation notes

1. Li^+ strongly binds organic carbonates, i.e., Li^+ in PC is actually present as $Li(PC)_{4-6}^+$.[3-5] Thus, $Li(PC)_4^+$ desolvation, reaction (RS1), could eventually control reduction and propagation rates and, hence, λ, if PC were consumed too rapidly.

$$Li(PC)_{4-6}^+ = Li(PC)_{3-5}^+ + PC \qquad \text{(RS1)}$$

2. Thus, while pure PC is 11.8 M, 1 M Li^+ solutions only contain ~ 6 M free PC in the bulk liquid. However, at the cathode surface, and later at the front of the advancing polymer Li^+ concentrations are enhanced by migration under the applied electric field. Because PC points its carbonyl C=O group to Li^+ in $Li(PC)_{4-6}^+$ but must point it to the electrode to capture an electron,[6] only free PC can undergo reduction, reaction R1, or participate in propagation, reaction R2.
3. The release of strongly bound PC is slow on account of its large endoergicity. The free energy of activation of reaction RS1, $\Delta G_{S1}^\dagger$, can be estimated from the time scales of the experiments of Figure 1 and transition state theory. From Figures 1A and B we infer that the half-life time of reaction RS1, $\tau_{1/2}$, is bracketed by the times required to scan ~ 0.4 V (i.e., from the onset of PCR up to peak currents at ~1.3 V, see above) at v = 0.5 mV s^{-1} and 5 mV s^{-1}: i.e., 800 s > $\tau_{1/2}$ > 80 s. Let us assume that $\tau_{1/2}$ ~ 400 s. Hence, from $k_{S1} = 0.69/\tau_{1/2} \sim 1.7\times10^{-3}\ s^{-1} = (k_B T/h) \times \exp(-\Delta G_{S1}^\dagger/RT) = (6.3 \times 10^{12}\ s^{-1}) \times \exp(-\Delta G_{S1}^\dagger/RT)$, ($k_B$, and h are Boltzmann's and Planck's constants. T = 300 K), we obtain $\Delta G_{S1}^\dagger \sim 22 \pm 2$ kcal mol^{-1}. If the reverse reaction (i.e., the binding of PC to Li^+) is barrierless, as expected. i.e.: $\Delta G_{-S1}^\dagger \sim 0$ kcal mol^{-1}, then $\Delta G_{S1} = \Delta G_{S1}^\dagger - \Delta G_{-S1}^\dagger \sim \Delta G_{S1}^\dagger = 22 \pm 2$ kcal mol^{-1}, which is in

excellent agreement with the ΔG_{S1} value recently reported by Othani et al., from quantum mechanical calculations.[3]

Polymerization notes –

1. Direct evidence that free organic radicals participate in these systems has been provided by electron paramagnetic resonance spectroscopy.[7,8]
2. Oligomers have been positively identified in SEI deposits by ex situ MALDI spectrometry.[9] Inorganic lithium oxides and (di)carbonates have been also detected therein.[10] Thus, on the basis of solid available experimental evidence, we assume that polymers are key components of SEI layers. It has been long realized[11] that given their respective mechanisms of formation, inorganic species, which largely derive from CO_2, are produced at initiation rates, r_i, whereas polymer mass increases at propagation rates, r_2, which may be significantly larger than r_1: $r_2 = \lambda\, r_1$, where λ is the degree of polymerization. By the same token,[11] inorganic species are localized in the deeper SEI layers adjacent to the electrode surface, whereas polymers will extend into the electrolyte.
3. By assuming that PC has a molecular diameter of 4 Å or a cross section of 1.3×10^{-15} cm^2 molecule^{-1}, Q = 0.65 mC cm^{-2} amounts to the reduction of ~ 5 PC molecules site^{-1}. Thus, a typical 20 nm thick SEI layer[11] corresponds to a material consisting of: $\lambda \sim 20\text{nm}/(0.4\text{nm} \times 5) = 10$, PC 10-mers on average.
4. Radical anion carriers $X^{\cdot-}$ may terminate by reacting with proton donor impurities, RH, such as H_2O, reaction RS2:

$$X^{\cdot-} + X^{\cdot-} \rightarrow X_2^{2-} \quad \text{(RS2)}$$

5. Note that if PC polymerization were driven by anions rather than free radicals, termination would occur via reaction RS2, with: $\lambda = \frac{k_2[PC]}{k_{S2}[IMP]}$. Thus, since the amount of impurities is fixed, once impurities were titrated polymerization would continue until all the solvent polymerizes. For example, < 1 mM H_2O impurities in a 20 nm interfacial layer would be titrated by the molar equivalent of Q ~ 20 μC cm^{-2} radical anions, which is 3-orders of magnitude smaller than those circulated in our experiments. In that case, λ would be a strong increasing function of circulated charge Q rather than of current density, at variance with our observations.
6. SEI formation actually involves which polymerization chemical reactions that take place under concentration and viscosity gradients, i.e., coupled to diffusive mass transfer. It has been shown that under such conditions the $\lambda > 1$ condition represents the onset of chain propagation along travelling waves.[12]

References

[1] S. Choudhury, L.A. Archer, Advanced Electronic Materials 2 (2016).
[2] Y.Y. Lu, Z.Y. Tu, L.A. Archer, Nature Materials 13 (2014) 961.
[3] H. Ohtani, Y. Hirao, A. Ito, K. Tanaka, O. Hatozaki, J. Thermal Anal. Calorim. 99 (2010) 139.
[4] K. Xu, Chem. Rev. 114 (2014) 11503.
[5] K. Xu, A.V. Cresce, J. Materials Res. 27 (2012) 2327.
[6] O. Borodin, Modern Aspects of Electrochemistry 58 (2014) 371.
[7] I.A. Shkrob, Y. Zhu, T.W. Marin, D. Abraham, J. Phys. Chem. C 117 (2013) 19255.
[8] I.A. Shkrob, Y. Zhu, T.W. Marin, D. Abraham, J. Phys. Chem. C 117 (2013) 19270.
[9] H. Tavassol, J.W. Buthker, G.A. Ferguson, L.A. Curtiss, A.A. Gewirth, J. Electrochem. Soc. 159 (2012) A730.
[10] H. Ota, Y. Sakata, A. Inoue, S. Yamaguchi, J. Electrochem. Soc. 151 (2004) A1659.
[11] E. Peled, J. Electrochem. Soc. 126 (1979) 2047.
[12] C. Bureau, J. Electroanal. Chem. 479 (1999) 43.
